# Stress–Strain and Structural Evolution on the Localized Interface of Stainless Steel Clad Plate

**DOI:** 10.3390/ma18143255

**Published:** 2025-07-10

**Authors:** Yinpeng Wang, Bo Gao, Qiqing Tian, Chunhui Jiang, Lu Zhu, Yanguang Cao, Wei Wei, Zhaodong Li

**Affiliations:** 1 School of Materials Science and Engineering, Changzhou University, Changzhou 213164, China; wangyinpeng2024@163.com (Y.W.); jch_617@163.com (C.J.); 2 Institute for Structural Steels, Central Iron & Steel Research Institute Company Limited, Beijing 100081, China; bjtugb@163.com (B.G.); tqqdut@163.com (Q.T.); zhulu5270@163.com (L.Z.); caoyanguang@cisri.com.cn (Y.C.); 3 Material Science & Engineering Research Center, School of Mechanical, Electronic and Control Engineering, Beijing Jiaotong University, Beijing 100044, China

**Keywords:** weathering steel–stainless steel clad plates, localized interfacial region, element diffusion, microstructural evolution, nanoindentation, stress–strain behavior

## Abstract

By applying different heat treatment processes (furnace cooling, air cooling, and water cooling), the stress–strain behavior of the localized interfacial region in weathering steel–stainless steel clad plates was investigated using nanoindentation, along with an analysis of interfacial microstructure formation and strengthening mechanisms. The results show that samples in the as-rolled (R), furnace-cooled (FC), air-cooled (AC), and water-cooled (WC) conditions exhibit distinct interfacial morphologies and local mechanical properties. A well-defined interfacial layer forms between the base and cladding materials, where a high density of dislocations, grain boundaries, precipitates, and nanoscale oxides significantly enhances interfacial strength, resulting in a yield strength (*R_p0.2_*) much higher than that of either adjacent metal. Across the transition from weathering steel to stainless steel, the interfacial region consists of ferrite—interfacial layer—“new austenite”—stainless steel austenite. Its formation is predominantly governed by element diffusion, which is strongly influenced by the applied heat treatment. Variations in diffusion behavior significantly affect the microstructural evolution of the dual-phase transition zone at the interface, thereby altering the local mechanical response.

## 1. Introduction

The yield strength of bridge steel has continuously evolved from 235 MPa to higher strength grades such as 345 MPa, 420 MPa, and beyond. In addition, increasingly complex service environments have raised higher demands for the weathering and corrosion resistance of bridge steels. Weathering steel–stainless steel clad plates, which combine the excellent corrosion resistance of stainless steel with the good toughness and low cost of carbon steel, offer promising application prospects in the field of bridge engineering [1]. In stainless steel clad plates, the presence of a bonded interface is one of the most defining and critical features [2,3,4]. This interface not only connects two metals with vastly different mechanical and chemical properties but also plays a decisive role in determining the structural performance and service reliability of the composite [5,6]. The quality of interfacial bonding, microstructural evolution, and mechanical behavior has long been a focal point of research. Li et al. [7] divided the interface of a 304 + Q235 stainless steel composite into the following three regions: a carburized layer on the stainless steel side, a diffusion layer, and a decarburized layer on the carbon steel side, with respective thicknesses of approximately 80 µm, 20 µm, and 150 µm. The high carbon content in the substrate can lead to severe decarburization, rendering the decarburized zone a significant stress concentration region during service. In addition, the diffusion zone has not been thoroughly investigated. Wang et al. [8] further found that, in 1Cr17/8Cr13MoV/1Cr17 multilayer composites, regions near the interface contained high densities of geometrically necessary dislocations (GNDs) and refined grains, which enhanced resistance to crack initiation. They suggested that Ni diffusion played a dominant role in the formation of this fine-grained interfacial structure. Liu et al. [9] conducted a detailed study on stainless steel clad plates composed of a Q235 carbon steel substrate and a 304 stainless steel cladding. They found that the uphill diffusion of carbon and the formation of concentration peaks at the interface resulted from the differences in diffusion rates and solubility of carbon between the substrate and cladding. The diffusion behavior of Cr and Ni was attributed to the combined effects of diffusion coefficients and concentration gradients. They conducted in-depth investigations on the diffusion zone and identified high dislocation densities and grain refinement at the interface. However, these studies lacked a detailed description of the structural formation and composition, and they failed to establish a direct correlation with the localized interfacial mechanical properties. Moreover, the high carbon content in the substrate may lead to significant carbon diffusion into the stainless steel, potentially triggering intergranular corrosion [10]. Zhang et al. [11] reported that, in 304 + Q235 composites, a high yield ratio of the interface was exhibited at the macro scale due to the contribution of the stainless steel, while at the micro scale, the interface exhibited higher yield strength dominated by the carbon steel side, highlighting the multiscale mechanical complexity of the interface. These findings underscore the challenges of characterizing and predicting interfacial behavior across different observation scales. In particular, establishing a direct correlation between elemental diffusion, microstructural evolution, and localized mechanical properties at the interface remains difficult, thereby complicating the prediction of macroscopic fracture behavior.

Nanoindentation has emerged as a powerful technique for evaluating mechanical properties at the micro- and nanoscales [12,13,14]. This method evaluates local hardness and stress–strain responses by analyzing the load–displacement curves, enabling high-resolution characterization of the local strength, elastic modulus, and hardness at phase boundaries, precipitation zones, and diffusion layers at the composite interface [15,16,17,18,19,20]. Compared to conventional macro-scale tensile or hardness tests, nanoindentation is particularly suited for probing localized strengthening mechanisms, the effects of element diffusion, and second-phase precipitation near complex interfacial regions.

Element diffusion near the interface inevitably leads to microstructural layering, with each layer exhibiting distinct characteristics and mechanical properties. Therefore, in this study, various heat treatment processes (FC, AC, WC) were applied to 316L + Q420qENH steel clad plates to obtain different interfacial states. The formation and evolution mechanisms of the interface were thoroughly investigated by introducing nanoindentation testing, and the gradient distribution and variation trends of interfacial strength under different conditions were characterized. Furthermore, the mechanical behavior of the localized interfacial region was systematically analyzed in relation to its underlying microstructural features. The findings provide both theoretical support and experimental validation for the future optimization of interfacial performance in clad steel plates.

## 2. Materials and Methods

### 2.1. Material and Testpiece

Q420qENH weathering bridge steel was selected as the base material and 316L stainless steel as the cladding material. The chemical compositions of the base and cladding materials are listed in Table 1. The plates were assembled via vacuum electron beam welding, followed by multi-pass hot rolling to produce a clad plate consisting of 316L + Q420qENH. The steel was produced in a vacuum induction furnace and forged into billets with 250 mm × 110 mm × 110 mm dimensions. After reheating to 1200 °C and homogenizing for 2 h, it was rolled to a final thickness of 19 mm, with rough rolling and finish rolling conducted at 1100 °C and 800 °C, respectively.

**Figure 1:**
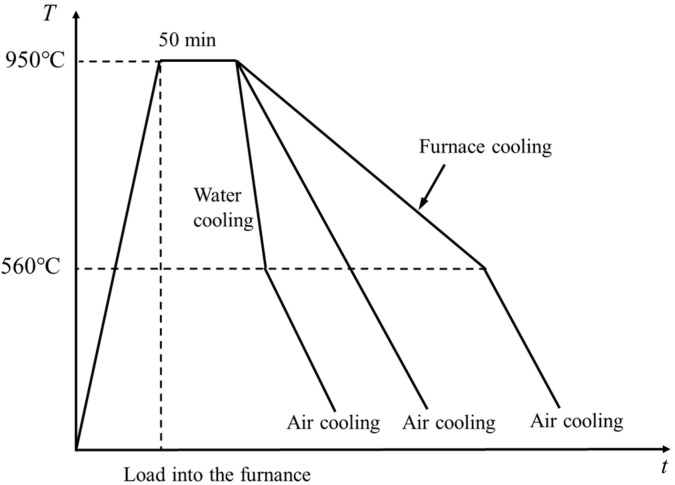
Heat treatment process diagram.

The as-rolled 316L + Q420qENH clad plates were subjected to austenitization heat treatment, followed by different cooling methods as shown in Figure 1. Based on the continuous cooling transformation (CCT) diagram of Q420qENH steel [21], the following three cooling strategies were employed: air cooling to room temperature, furnace cooling to 560 °C followed by air cooling to room temperature, and water cooling to 560 °C followed by air cooling to room temperature. The average cooling rates were 0.25 °C/s (furnace cooling), 1 °C/s (air cooling), and 50 °C/s (water cooling), and the water cooling process was conducted in a repetitive “water quenching–rapid removal–temperature measurement” manner, typically requiring 3 to 4 cycles to lower the specimen surface temperature to approximately 560 °C. Clad plates that underwent different heat treatments were named FC sample, AC sample, and WC sample, respectively. The as-rolled 316L + Q420qENH clad plate was named the R sample.

### 2.2. Testing Method

Cross-sectional metallographic samples (5 mm in length) were prepared from the experimental steels using wire electrical discharge machining (EDM). Samples were pre-ground using 150#, 320#, 600#, and 1000# grit SiC papers, followed by fine polishing until scratch-free surfaces were obtained. The polished surfaces were etched in a 4 vol.% nitric acid ethanol solution for 8~10 s, rinsed with ethanol, and dried. Microstructural characterization was performed using an optical microscope (OM) and a scanning electron microscope (SEM). Elemental distribution in the localized interfacial region of etched composite samples was analyzed qualitatively and quantitatively using a Shimadzu EPMA-1720H electron probe microanalyzer (SHIMADZU Corporation, Kyoto, Japan), with a step size of 0.5 μm. Electrolytic polishing was conducted using a 10 vol.% perchloric acid and 90 vol.% anhydrous ethanol solution, with a Buehler ElectroMet 4 electropolisher (Buehler, Lake Bluff, IL, USA). Microstructural observation was carried out using a Thermo Scientific Apreo 2C field emission SEM equipped with a Velocity™ EBSD detector (Oxford Instruments NanoAnalysis, High Wycombe, UK). Grain size and low-angle grain boundary fractions were statistically analyzed using AZtec Crystal (2.1.259) software, with a scan step size of 0.2 μm. Focused ion beam (FIB) milling at the interface was performed using a Zeiss Crossbeam 550 system (Carl Zeiss Microscopy GmbH, Oberkochen, Germany). The thin lamella (6 μm depth × 8 μm length × 2 μm width) was thinned to a uniform thickness of 60–70 nm. A tungsten protective layer and a copper lift-out grid were used. Morphology and elemental analysis were conducted with an FEI Talos F200X (Thermo Fisher Scientific, Hillsboro, OR, USA) transmission electron microscope (TEM).

**Figure 2:**
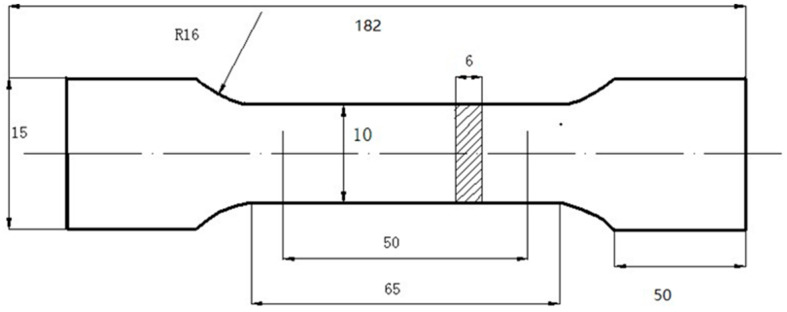
Tensile test specimens (unit: mm).

Nanoindentation tests were conducted using a Nano Indenter G200 system (Keysight Technologies, Santa Rosa, CA, USA) with a Berkovich tip. The tests employed a depth-controlled method with continuous stiffness measurement (CSM) [22], setting the maximum indentation depth to 200 nm. The strain rate was 0.05 s^−1^, harmonic displacement 2 nm, frequency 45 Hz, and loading rate 10 nm/s. The sampling of tensile specimens was as follows: starting from the stainless steel side, a 6 mm thick specimen was taken with stainless steel and weathering steel, each accounting for 3 mm, and then processed into tensile test specimens (according to GB/T 228.1-2021 [23]) as shown in Figure 2, the loading speed was set to 2 mm/min, and two parallel specimens were tested under each condition.

**Figure 3:**
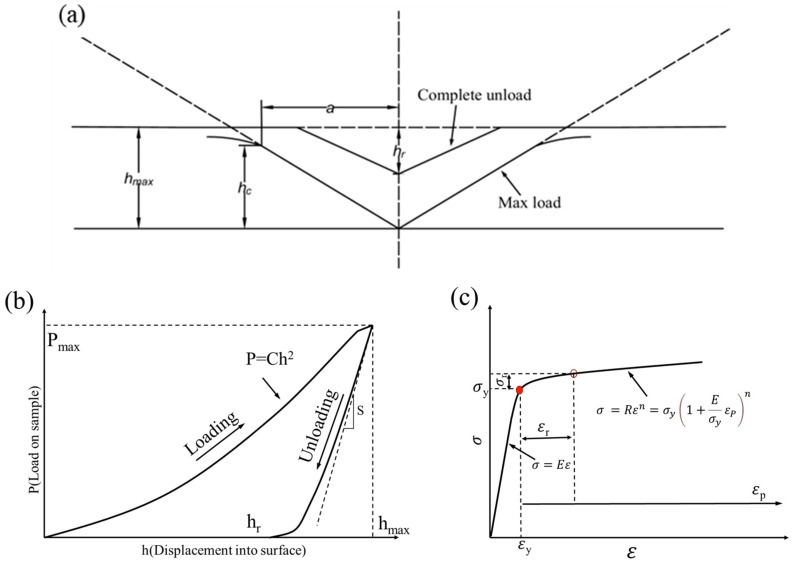
(**a**) Schematic of the indentation shape of the Berkovich indenter; (**b**) schematic of a typical load–depth curve; (**c**) schematic of the power law elasto-plastic stress–strain behavior.

Figure 3a shows a schematic diagram of the indentation shape of the Berkovich indenter. Based on the specific shape of the Berkovich indenter (equivalent half cone angle of 70.3°), the relationship between the actual contact area A_c_ and the depth of indentation contact *h_c_* (the definitions of the variables in Equation (1) are shown in Figure 3b) exist in the ideal case as follows [24]:

(1) $$A_{c}=24.56h_{c}^{2}=\frac{P_{max}}{H}$$

By the Oliver and Pharr method [19,24]:

(2) $$P_{u}=B{\left(h-h_{r}\right)}^{m}$$

(3) $${\left.\frac{{dP}_{u}}{dh}\right|}_{h_{max}}={Bm\left(h_{max}-h_{r}\right)}^{m-1}$$

where (3) is derived from Equation (2); $h_{max}\;$ is the maximum indentation depth; $P_{u}$ is the load at the beginning of unloading; *B* is the fitting parameter; *H* is the average contact pressure, i.e., the hardness; *m* is the shape parameter of the indentation, and the value of m changes according to the different indentation materials for the Berkovich indenter and is usually 1~1.5; *B* is the fitting parameter, and the fitting range is selected as follows: the initial unloading point in the upper 25~50% of the unloading curve is used to determine the best fitting parameters.

The relationships between the discount modulus *E_r_* and the modulus of elasticity $E_{i}$ of the indenter, Poisson’s ratio and the modulus of elasticity *E*, and Poisson’s ratio *v* of the specimen exist as follows [25]:

(4) $$\frac{1}{E_{r}}=\frac{1-v^{2}}{E}+\frac{1-v_{i}^{2}}{E_{i}}$$

where *E_i_* and *v_i_* are the modulus of elasticity of the indenter and Poisson’s ratio, and for the Berkovich diamond indenter, they were 1140 GPa and 0.07; E and v are the modulus of elasticity of the material and Poisson’s ratio; the stainless steel and low-alloyed steel specimen Poisson’s ratio is 0.3.

Meanwhile, for the initial unloading period, the discounted reduction modulus *E_r_* can also be derived from $A_{c}$ and ${\left.\frac{{dP}_{u}}{dh}\right|}_{h_{max}}$, as follows [26]:

(5) $$E_{r}={\left.\frac{1}{c^{*}\sqrt{A_{c}}}\frac{{dP}_{u}}{dh}\right|}_{h_{max}}$$

In the formula, for the elastic deformation under the Berkovich indenter at initial unloading, c* = 1.167, the elastic modulus *E* and the discount modulus *E_r_* of the specimen microregion can be obtained by coupling Equations (4) and (5), and then the microregion stress–strain relationship can be further obtained, and a series of quantitative analysis methods proposed by Dao [15] et al. can be used to obtain the complete microregion stress–strain relationship (Figure 3c shows the schematic diagram of the power law elasto-plastic stress–strain behavior):

(6) $$\sigma =\left\{\begin{array}{c} E\varepsilon \;\;\;\;\;\;\;\;\;\;\;\;\;\;\;\;\;\;\;\;\;\;\;\;\;\;\;\;\;\;\;\;\;\;\;\;\;\;\;\;\;\;\;\;\;\;\;\;\;\;\;\;\;\left(\sigma \leq \sigma_{y}\right)\\ R\varepsilon^{n}=\sigma_{y}{\left(1+\frac{E}{\sigma_{y}}\varepsilon_{P}\right)}^{n}\;\;\;\;\;\;\;\;\;\;\;\;\;\;\;\;\;\;\;\left(\sigma >\sigma_{y}\right) \end{array}\right.$$

Since the relative stress is independent of the hardening index *n* at a relative strain *ε_r_* of 0.033, while the loading curvature *C* is obtained by direct fitting of the loading curve according to the Kick model [15], $\sigma_{0.033}$ can be solved according to the following Equation (7):

(7) $$\prod_{1}\left(\frac{E_{r}}{\sigma_{r}},n\right)=\frac{C}{\sigma_{r}}\to \prod_{1}\left(\frac{E_{r}}{\sigma_{0.033}}\right)=\frac{C}{\sigma_{0.033}}$$

Associatively, Equations (7) and (8) can be solved for *n*. Finally, the value of the yield strength *σ_y_* is calculated based on Equation (9), and finally, the stress–strain behavior of the material microregion is obtained based on Equation (6), as follows:

(8) $$\prod_{2}\left(\frac{E_{r}}{\sigma_{0.033}},n\right)={\left.\frac{1}{E_{r}h_{max}}\frac{{dP}_{u}}{dh}\right|}_{h_{max}}$$

(9) $$\sigma_{0.033}={\sigma_{y}(1+\frac{E}{\sigma_{y}}0.033)}^{n}$$

where $\prod_{1},\;\;\prod_{2}\;$ are dimensionless equations given by Dao [15] et al. All parameters mentioned above are determined through curve fitting using OriginPro 2024 (Learning Edition), and subsequent equation-based calculations were performed in MATLAB 2020a.

## 3. Experimental Results

### 3.1. Microstructure

**Figure 4:**
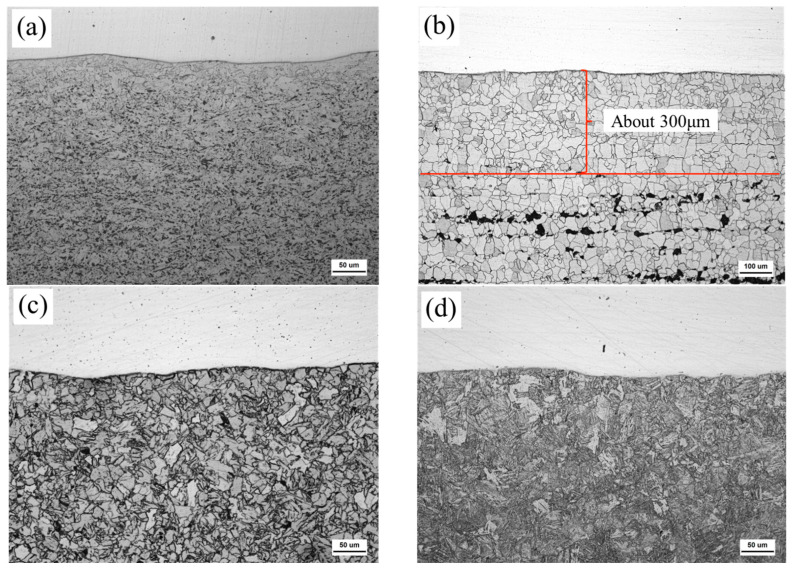
OM images near the interface of R (**a**), FC (**b**), AC (**c**), and WC (**d**) samples.

Figure 4 shows the microstructure near the interface and in the weathering steel matrix (away from the interface) of 316L + Q420qENH clad plates in the as-rolled state (R) and after FC, AC, and WC heat treatments. The stainless steel side remains unetched, showing a homogeneous austenitic structure. A dark line at the interface is visible under optical microscopy in the R, FC, and AC samples, but it becomes indistinct in the WC condition.

**Figure 5:**
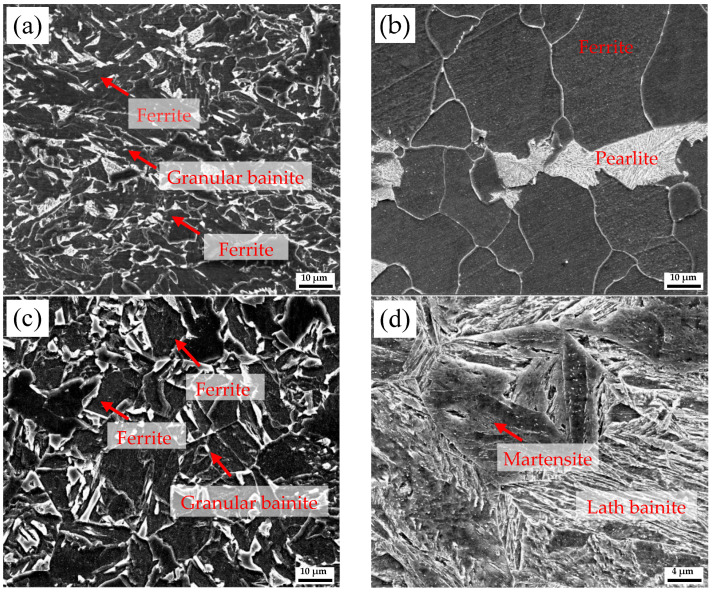
SEM images of the Q420qENH side in (**a**) R, (**b**) FC, (**c**) AC, and (**d**) WC samples.

Figure 5 shows the microstructure in the weathering steel matrix (away from the interface) of R, FC, AC, and WC samples. In the R sample, a ~20 μm ferrite layer is observed near the interface (Figure 3a), while the matrix consists of granular bainite and ferrite. In the FC sample, the interface-adjacent region contains a ~300 μm thick ferrite layer (Figure 3b), and the matrix comprises ferrite and pearlite. In the AC sample, the structure is ferrite plus granular bainite. In the WC sample, a mixture of martensite and lath bainite is observed.

**Figure 6:**
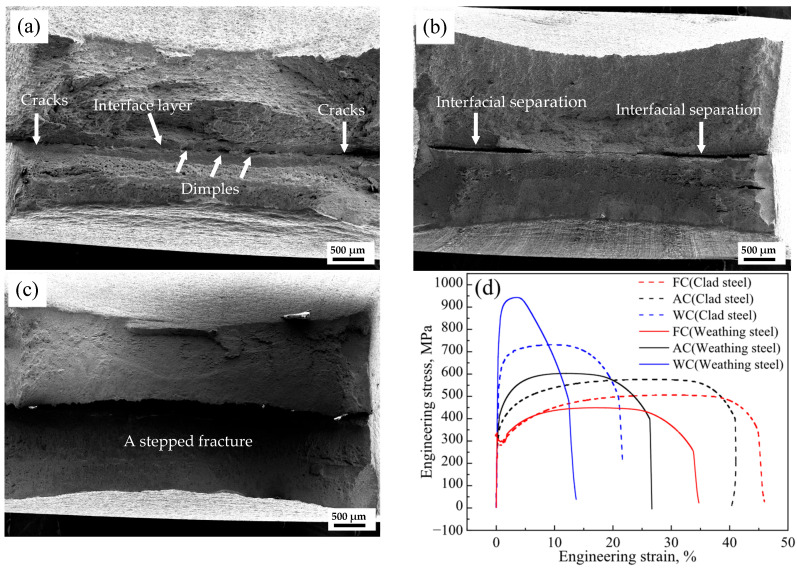
SEM images of tensile fracture: (**a**) FC, (**b**) AC, and (**c**) WC samples; (**d**) the stress–strain curves of clad steel and weathering steel.

Tensile tests were conducted in accordance with GB/T 228.1-2021, and the interfacial tensile features of FC, AC, and WC specimens are shown in Figure 6. The FC specimen was characterized by edge-proximal cracking and central dimples. The AC specimen showed interfacial delamination near both sides along with central cracking. In contrast, the WC specimen displayed a stepped fracture morphology. Macroscopic tensile results revealed that the interfacial fracture modes vary under different cooling conditions; the FC sample exhibited ductile fracture, while the AC and WC samples showed brittle cleavage fracture. These differences suggest that different heat treatment conditions may result in distinct interfacial states. Therefore, this study further investigates the stress–strain behavior and microstructural evolution in the localized interfacial region.

As shown in Figure 6d, for clad steel samples, yield strength (YS) and ultimate tensile strength (TS) increased with cooling rate (FC → AC → WC), reaching 282, 320, 584 MPa, and 505, 576, 731 MPa, respectively; elongation (EL) decreased from 47.5% to 43.75% and 24.00%. Weathering steel exhibited a similar trend in strength and ductility. Compared to Weathering steel, clad steel of FC increased 12%, clad steel of AC decreased 4.5%, and clad steel of WC reduced 23% in UTS. In the AC and WC samples, the microstructure away from the interface primarily consisting of bainite provided significant strengthening.

### 3.2. Nanoindentation

**Figure 7:**
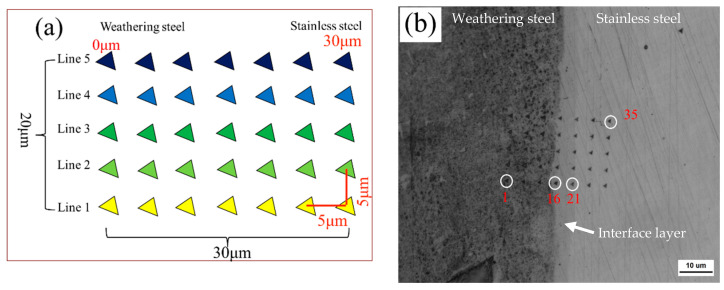
(**a**) Schematic diagram of nanoindentation point array; (**b**) OM image of nanoindentation points after slight etching.

As shown in Figure 7a, nanoindentation was performed from weathering steel to stainless steel near the interface of the R sample using a 5 × 7 grid, covering an area of 20 μm × 30 μm. Figure 7b displays the actual indentation array, starting from point “1” at the lower left and ending at point “35”, with a total of 35 indents. For the FC, AC, and WC samples, nanoindentation was conducted along Line 1, Line 2, Line 3, and Line 4 only.

**Figure 8:**
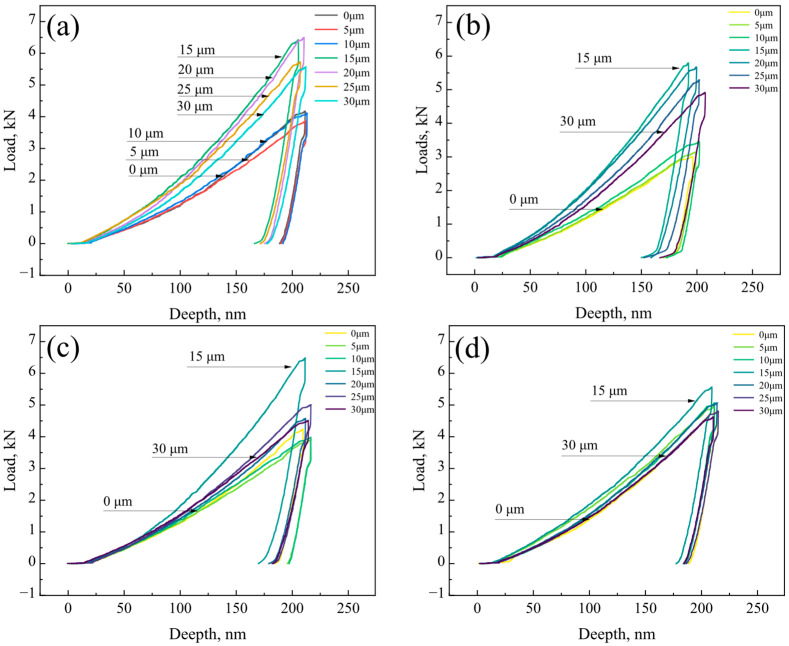
Load–depth curves of the localized interfacial region: (**a**) R, (**b**) FC, (**c**) AC, and (**d**) WC samples.

As shown in Figure 8, load–depth curves for the R, FC, AC, and WC samples illustrate the loading and unloading behavior during nanoindentation. Notably, peak loads consistently occurred at 15 or 20 μm, corresponding to the composite interface. The peak loads for the R, FC, AC, and WC samples were 6.49, 5.78, 6.48, and 5.55 mN, respectively. For the R, FC, and AC samples, the peak load followed the following trend: interface (~6 mN) > stainless steel side (~5 mN) > weathering steel side (3~4 mN). In contrast, for the WC sample, while the interface still exhibited the highest load, the weathering steel and stainless steel sides showed similar peak loads, both ranging from 4 to 5 mN.

**Figure 9:**
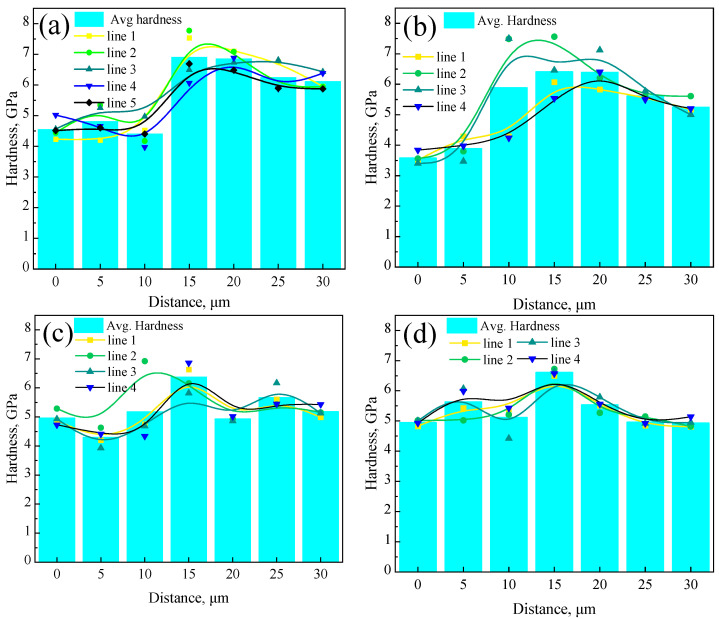
Hardness distribution of the localized interfacial region for different samples: (**a**) R, (**b**) FC, (**c**) AC, and (**d**) WC samples.

As shown in Figure 9, the bar charts display the average hardness distribution from the weathering steel side to the stainless steel side for the R, FC, AC, and WC samples. All four samples exhibited the following common trend: hardness increased from the weathering steel side, reached a peak at the interface, and then decreased toward the stainless steel side. The peak hardness occurred within 15~20 μm for the R sample, 10~20 μm for the FC sample, and at 15 μm for both the AC and WC samples.

**Figure 10:**
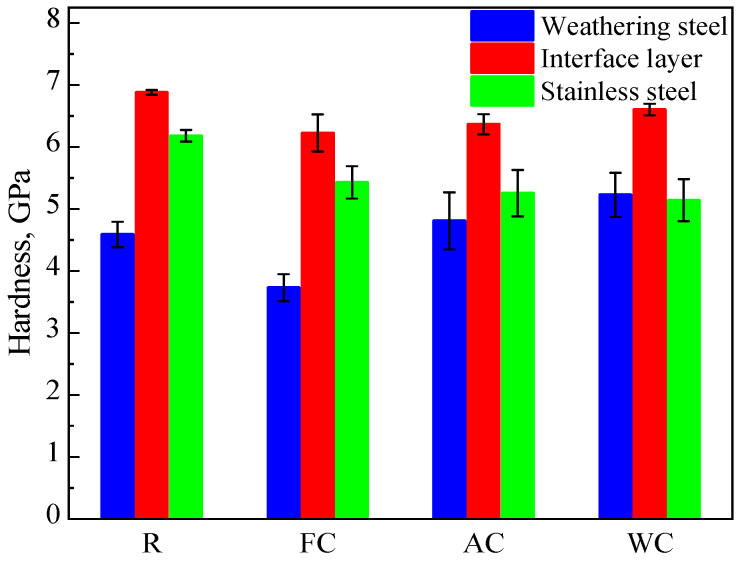
Hardness distribution in different regions of R, FC, AC, and WC samples.

As shown in Figure 10, the average hardness values across different regions of the R, FC, AC, and WC samples are presented. The average hardness values on the weathering steel side were 4.59, 3.73, 4.80, and 5.23 GPa for the R, FC, AC, and WC samples, respectively. At the interface, the average hardness values were 6.88, 6.23, 6.37, and 6.61 GPa, while on the stainless steel side, they were 6.18, 5.43, 5.26, and 5.14 GPa, respectively.

## 4. Discussion

### 4.1. Stress–Strain Analysis of the Localized Interfacial Region

Table 2 lists the parameters involved in the local stress–strain calculations for the R sample. The nanoindentation data were nondimensionalized and analyzed following the outlined procedure. The parameter *C* was obtained by fitting the loading portion of the curve in Figure 3b, while *E_r_*, *E*, *σ*_0.033_, *n*, *σ_y_*, and *ε_y_* were derived using fitting functions or calculated based on Equations (1)–(9). The same procedure was applied to the other samples, and their data are not listed here for brevity.

**Figure 11:**
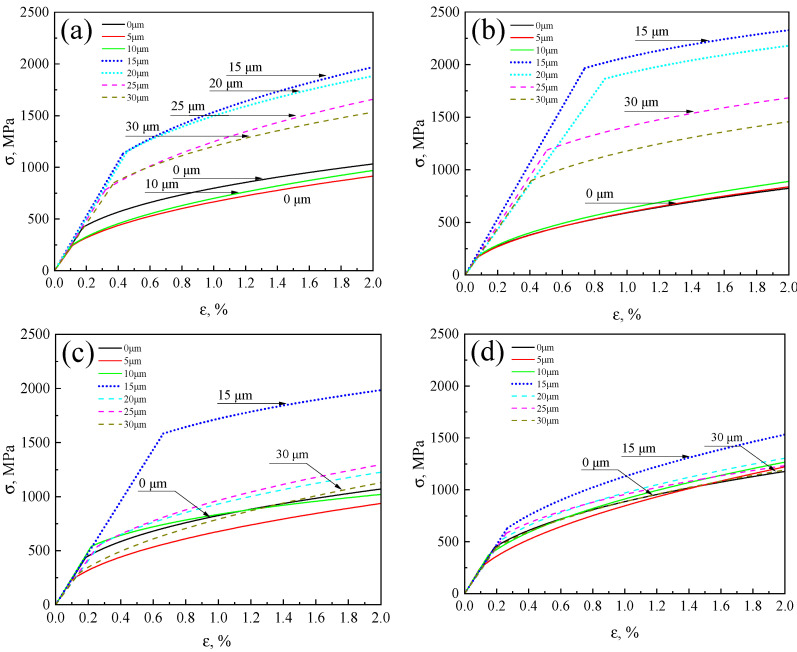
Local stress–strain curves: (**a**) R, (**b**) FC, (**c**) AC, and (**d**) WC samples.

Based on these parameters, the local stress–strain curves of the R, FC, AC, and WC samples were plotted, as shown in Figure 11. It can be observed that, in the R, FC, and AC samples, the stress–strain responses varied significantly across different regions (weathering steel side, interfacial layer, and stainless steel side). In contrast, the WC sample exhibited similar stress–strain behavior across all regions.

**Figure 12:**
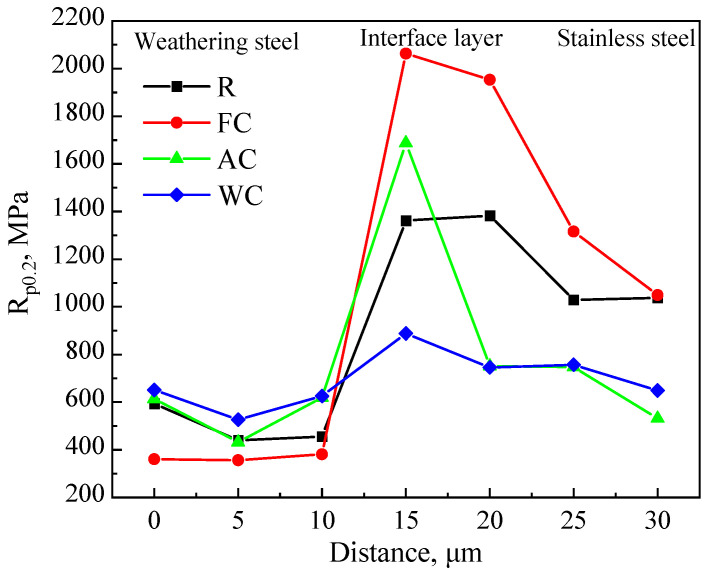
*R_p0.2_* curves of the localized interfacial region for R, FC, AC, and WC samples.

Based on the stress–strain curves, the 0.2% offset yield strength (*R_p0.2_*) was calculated and is shown in Figure 12. The *R_p0.2_* values of the interfacial layers were higher than those of the adjacent metals, reaching 1361.97 MPa (R), 2062.93 MPa (FC), 1687.68 MPa (AC), and 888.45 MPa (WC), respectively. On the weathering steel side, with an increasing cooling rate (from FC to AC to WC), the microstructure transitioned from F + P and F + Gra-B to Gra-B + Lath-B, accompanied by an increase in dislocation density. Accordingly, *R_p0.2_* increased from 360.74 MPa (FC) to 613.80 MPa (AC) and 650.90 MPa (WC). In the R sample, although the region near the interface (about 20 μm) consisted mainly of F (Figure 3a), grain refinement and dislocation strengthening induced by two-stage rolling resulted in an *R_p0.2_* of 593.26 MPa. On the stainless steel side, since the austenitic structure was unaffected by cooling rate and lost rolling-induced strengthening after austenitization, the strengths of the AC and WC samples were comparable, at 747.31 MPa and 756.86 MPa, respectively. In contrast, the R sample retained the strengthening effect introduced by rolling, resulting in a higher *R_p0.2_* of 1037.52 MPa [27,28,29].

### 4.2. Interfacial Structure

**Figure 13:**
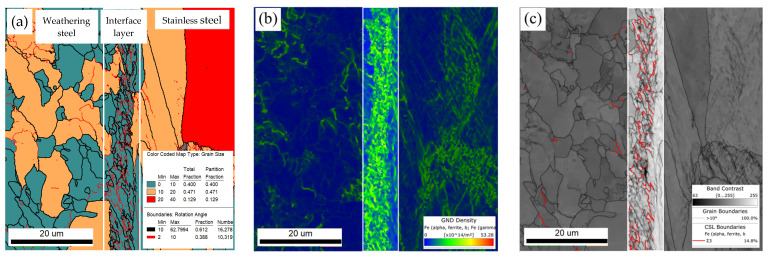
EBSD analysis of the localized interfacial region in R samples: (**a**) grain boundary (GB) distribution; (**b**) geometrically necessary dislocation (GND) distribution; (**c**) twin boundaries (TB) distribution.

Figure 13 shows the interfacial structure of the R sample obtained via EBSD. A transition layer approximately 6 μm thick is observed at the interface, consisting of the interfacial microstructure and adjacent zones on both the base and cladding sides, effectively bridging the two materials.

**Figure 14:**
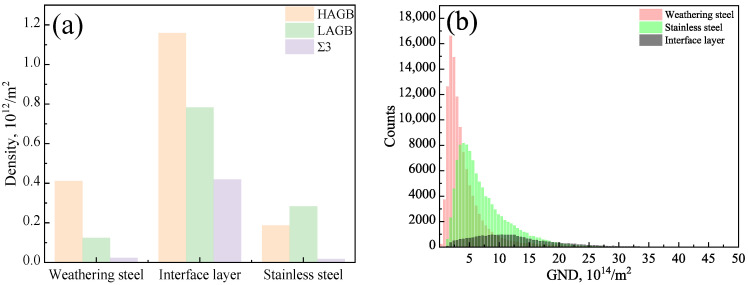
EBSD data analysis of the localized interfacial region in R samples: (**a**) GB distribution; (**b**) GND distribution.

The measured dislocation and grain boundary densities are summarized in Table 3 and shown in Figure 14a,b. Quantitative analysis of the EBSD data using AZtecCrystal software revealed that, within the interfacial layer, the high-angle grain boundary (HAGB) density was 1.16 × 10^12^ /m^2^, low-angle grain boundary (LAGB) density was 0.78 × 10^12^ /m^2^, TB density was 0.42 × 10^12^ /m^2^, and the GND density reached 12.29 × 10^14^ /m^2^. All of these values were significantly higher than those in the adjacent weathering steel and stainless steel regions.

**Figure 15:**
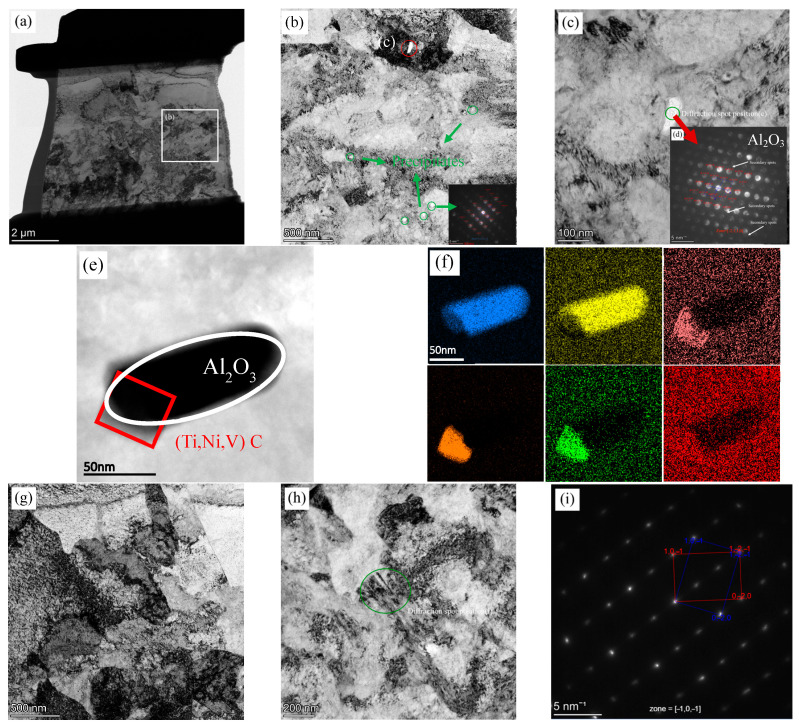
TEM analysis of the interface layer in R samples: (**a**) overall morphology; (**b**,**c**,**e**) inclusion distribution; (**d**) corresponding diffraction patterns of (**c**); (**f**) EDS elemental maps; (**g**) dislocations; (**h**) twin structures; and (**i**) their diffraction pattern.

As shown in Figure 15, TEM was used to examine the interfacial structure. Nanoscale inclusions are observed at the interface in Figure 15b,c,e. Elemental analysis of the nanoscale inclusion in Figure 15c was performed using energy-dispersive spectroscopy (EDS), with the elemental distributions shown in Figure 15f. The enrichment of Al and O, combined with the hexagonal close-packed (HCP) diffraction pattern identified in Figure 15d, confirmed that the primary phase of the inclusion was Al_2_O_3_. Based on the presence of Ti, Nb, V, and C, the second set of diffraction spots in Figure 15d was preliminarily identified as (Ti, Nb, V)C precipitates. Given that oxide forms at a high temperature, it is inferred that the precipitate nucleates and grows heterogeneously on the Al_2_O_3_ particle. Nanoscale Al_2_O_3_ inclusions and (Ti, Nb, V)C precipitates can further enhance interfacial strength through dislocation shearing or bypass mechanisms. The nanoscale inclusions and precipitates observed in this region may contribute to a certain degree of interfacial strengthening [30].

Additionally, dislocation structures observed in EBSD are confirmed in Figure 15g, along with twin structures shown in Figure 15h. The SAED pattern of the twins, displayed in Figure 15i, is indexed as a body-centered tetragonal (BCT) lattice, indicating martensitic twins.

**Figure 16:**
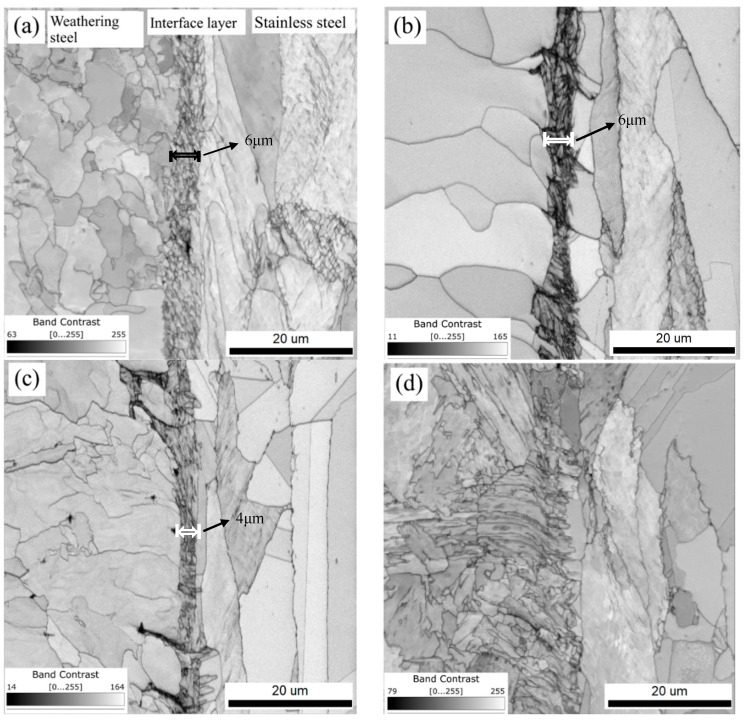
The EBSD band contrast images of the localized interfacial region: (**a**) R, (**b**) FC, (**c**) AC, and (**d**) WC samples.

As shown in Figure 16, EBSD band contrast images of the R, FC, AC, and WC samples revealed the microstructural transition from Q420qENH weathering steel to 316L stainless steel. In the FC, AC, and WC heat-treated samples, a distinct interfacial microstructure was observed in the transition zone between the two materials. Across all samples (R, FC, AC, WC), the interfacial layer exhibited higher grain boundary density, dislocation density, and twin density than either adjacent material. The widths of the interfacial substructure regions were approximately 6, 6, and 4 μm for the R, FC, and AC samples, respectively, and it was difficult to observe similar interface structures in WC samples. The presence of nanoscale inclusions such as Al_2_O_3_, precipitates like (Ti, Nb, V) C, high-density grain boundaries, and dislocations within the interface contributes to interfacial strengthening through multiple mechanisms, which are, namely, precipitation strengthening, grain boundary strengthening, and dislocation strengthening [31,32,33,34,35]. As a result, the yield strength at the interface is significantly higher than that of the adjacent regions (Figure 12). This strengthening effect simultaneously led to a transition in fracture mode, as shown in Figure 5, from the dimpled and cracked morphology observed in the FC sample to partial interfacial delamination in the AC sample, and ultimately to the stepped interfacial fracture seen in the WC sample.

### 4.3. Formation and Evolution of Interfacial Structure

**Figure 17:**
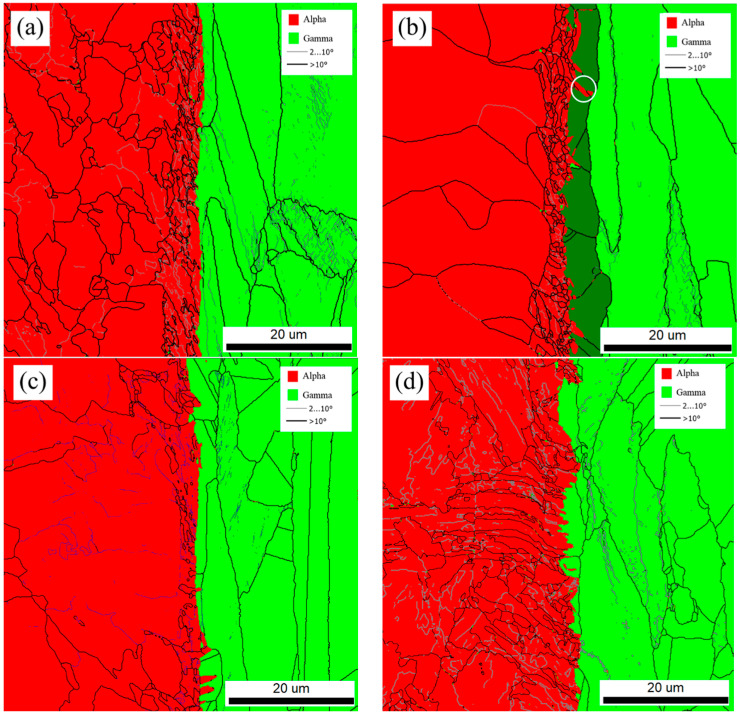
EBSD phase maps of the localized interfacial region: (**a**) R, (**b**) FC, (**c**) AC, and (**d**) WC samples.

As shown in Figure 17, EBSD phase maps of the R, FC, AC, and WC samples revealed distinct interfacial features. In all four samples, numerous small protrusions of the alpha (α) phase extended into the adjacent gamma (γ) phase near the interface, often aligned along grain boundaries. These protrusions were most prominent in the FC sample (Figure 17b), with some penetrating as deep as 2.8 μm. Since the primary difference among FC, AC, and WC was the cooling rate, these α-phase intrusions into the γ-phase were inferred to form during the cooling stage. Additionally, the morphology of austenite grains near the interface differed from that in regions farther away. For example, in Figure 17b, as highlighted in dark contrast, the interfacial A grains exhibited features more closely resembling the F grains in weathering steel.

**Figure 18:**
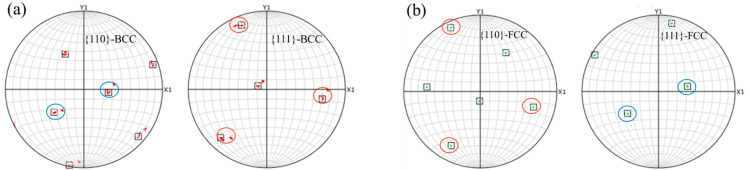
Pole figures of the marked region in the FC sample: (**a**) alpha-phase protrusion; (**b**) adjacent gamma-phase above.

As shown in Figure 18, pole figures were generated for the alpha-phase protrusion and the adjacent gamma-phase region above it. The <110> and <111> pole figures were obtained by analyzing the EBSD data from the white-circled alpha-phase protrusion and the adjacent dark gamma-phase in Figure 18b using AZtecCrystal software. The analysis revealed a Kurdjumov–Sachs (K–S) orientation relationship between the two grains, specifically {111}γ ∥ {110}α [36,37]. This indicates that the dark gamma-phase grain in Figure 18b shares not only a similar morphology with the adjacent gamma grain on the left but also exhibits parallel crystallographic planes.

**Figure 19:**
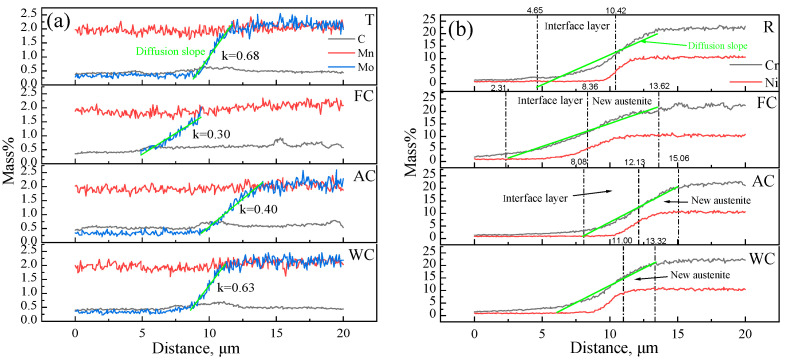
Elemental line scans of the localized interfacial region: (**a**) C, Mn, and Mo; (**b**) Cr and Ni.

As shown in Figure 19, elements such as C, Mn, Mo, Ni, and Cr exhibited varying degrees of diffusion across the interface in the R, FC, AC, and WC samples. The extent of elemental enrichment near the phase boundary differed under different cooling conditions. For a given concentration gradient, each element showed a distinct diffusion length near the interface. For Cr, within the 5–20% concentration range, the diffusion length in the R sample was 6.9 μm, while for FC, AC, and WC samples, it increased to 11.5, 7.0, and 7.3 μm, respectively. For Mo (0.3–2.0%), the diffusion lengths were 2.9 μm for R, and 4.5, 4.6, and 2.6 μm for FC, AC, and WC, respectively. For Ni (0.3–10.0%), the diffusion lengths were 1.4 μm for R, and 5.3, 4.6, and 3.2 μm for FC, AC, and WC, respectively. To quantify this behavior, linear fitting was applied to the element concentration profiles using Origin software within the specified gradient range. The slope (*k*) of the fitted line was used to characterize interfacial diffusion, where a smaller *k* value indicates a greater degree of diffusion.

The diffusion gradient curves of Cr, Ni, and Mo were linearly fitted, and the corresponding diffusion lengths and slopes for each sample are summarized in Table 4. It can be observed that the FC sample exhibited the greatest diffusion extent, indicating higher long-range solute diffusion of Cr, Ni, and Mo at the interface. Combined with the previously noted similarities in grain morphology and the presence of a K–S orientation relationship, the dark γ phase was identified as “new austenite” formed by the solid solution of Cr, Ni, and Mo during the holding and cooling stages. In previous experimental studies under varying vacuum levels [38], it was observed that the interfacial oxides formed during rolling remained within the austenite matrix after cooling, providing indirect evidence for the formation of “new austenite”.

**Figure 20:**
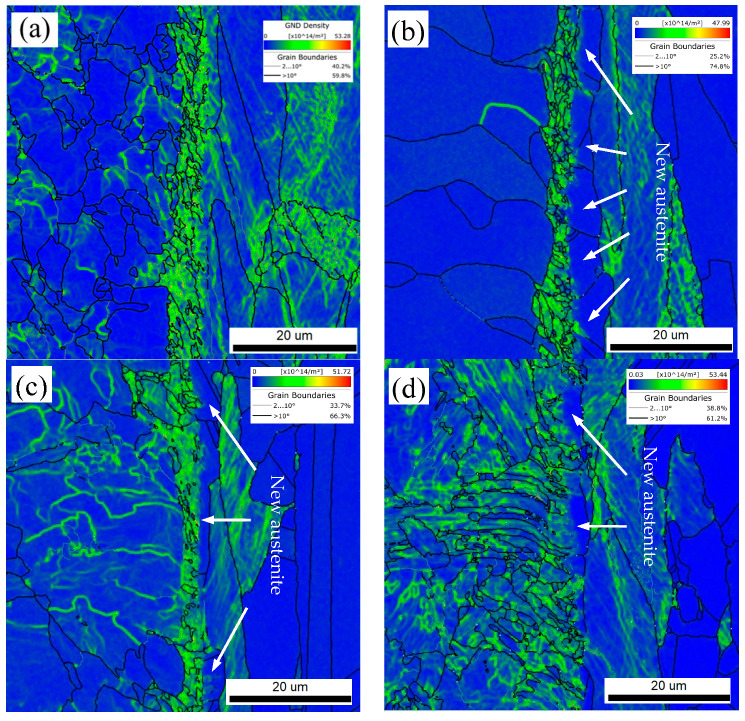
GND and new austenite of the localized interfacial region: (**a**) R, (**b**) FC, (**c**) AC, and (**d**) WC samples.

As shown in Figure 20, the distributions of GND and GB in the R, FC, AC, and WC specimens revealed the interfacial microstructures in detail. Compared with the phase maps in Figure 18, it can be observed that, after the FC, AC, and WC heat treatments, the GND density in the “new austenite” region was reduced to nearly zero. In the FC specimen, the “new austenite” layer was the thickest, with an alloy diffusion length of approximately 11.5 μm, closely matching the sum of the interfacial substructure layer (~6 μm) and the “new austenite” layer (~6 μm). A similar diffusion profile was observed in the AC specimen.

These observations indicate that both the “new austenite” and the substructure layer are formed through extensive solid solution of alloying elements such as Cr, Ni, and Mo during high-temperature exposure and subsequent cooling. During the cooling process, the interface between the “new austenite” and the substructure layer serves as a phase transformation boundary. When the local solid solution level exceeds this critical threshold, the retained austenite remains stable at room temperature. Conversely, when the concentration falls below this threshold, the metastable austenite formed under slow cooling conditions (0.25–1 °C/s) may develop a high density of GNDs, possibly due to factors such as transformation-induced stress and lattice distortion. This promotes further grain refinement and contributes to the formation of a rough and interlocking morphology at the phase boundary.

## 5. Conclusions

In this study, the rolled clad steels were subjected to austenitization followed by furnace cooling, air cooling, and water cooling, resulting in R, FC, AC, and WC samples. A systematic investigation was conducted on the interfacial morphology and the local stress–strain response at the interface to elucidate the formation and strengthening mechanisms of the localized interfacial region. The findings provide theoretical support and experimental data for practical production and process optimization. The main conclusions are as follows:

(1) The interfacial layer consistently exhibited higher strength than both adjacent metals and was observed in the R, FC, and WC samples. The interfacial layer is characterized by significantly higher densities of grain boundaries, dislocations, and twin boundaries and is further reinforced by nanoscale inclusions and precipitates. These features collectively contribute to grain boundary strengthening, dislocation strengthening, and second-phase strengthening. Specifically, in the R sample, the densities of HAGBs, LAGBs, TBs, and GNDs in the interfacial layer reach 1.16 × 10^12^/m^2^, 0.78 × 10^12^/m^3^, 0.42 × 10^12^/m^2^, and 2.29 × 10^14^/m^2^, respectively, which are substantially higher than those observed on both the weathering steel side (0.4117, 0.124, 0.02395, 4.12 × 10^14^/m^2^) and the stainless steel side (0.1869, 0.28372, 0.0177, 7.28 × 10^14^/m^2^).

(2) Across the transition from weathering steel to stainless steel, the interfacial region consists of ferrite—interfacial layer—new austenite—stainless steel austenite. Due to the significant compositional differences between them, elements such as Cr, Ni, and Mo diffuse across the interface to varying extents. During the cooling process, The ferrite near the interface is formed through a ferritic transformation induced by Cr solid solution. When the local solid solution level exceeds this critical threshold, the retained austenite remains stable at room temperature. Conversely, when the concentration falls below this threshold, the metastable austenite formed under slow cooling conditions (0.25–1 °C/s) may develop a high density of GNDs, possibly due to factors such as transformation-induced stress and lattice distortion. This promotes further grain refinement and contributes to the formation of a rough and interlocking morphology at the phase boundary. With an increasing cooling rate (from FC to AC to WC), the thicknesses of the new austenite, substructure region, and interfacial ferrite layer all decrease.

(3) Stress–strain analysis from nanoindentation reveals that interfacial strength is closely related to the evolution of interfacial microstructure. The cooling rate affects both element diffusion and the transformation of metastable austenite. Using 1/*k* (inverse slope of diffusion gradient) to quantify diffusion extent, the order for Cr, Ni, and Mo is shown as follows: FC (0.54, 0.578, 3.33) > AC (0.36, 0.46, 2.50) > WC (0.36, 0.31, 1.60). This trend corresponds to the thickness of the interfacial layer substructure region (FC > AC > WC) and ultimately results in the following interfacial yield strengths (*R_p0.2_*): 2062.93 MPa (FC), 1687.68 MPa (AC), and 888.45 MPa (WC). Slower cooling promotes more complete diffusion and phase transformation, leading to a stronger interface. Thus, the FC specimen showed a dimpled fracture with minor cracking, outperforming the interfacial delamination in AC and the stepped fracture in WC.

## Figures and Tables

**Table 1:** Chemical composition of 316L and Q420qENH (wt.%).

Element	C	Si	Mn	Cu	Cr	Ni	Mo	Al	Ti	Nb	V
Q420qENH	0.057	0.22	1.31	0.28	0.45	0.32	0.058	0.026	0.014	0.029	0.03
316L	0.027	0.53	1.19	/	16.78	10.39	1.99	/	/	/	/

**Table 2:** Computational parameters involved in the process of the local stress–strain calculation of the R sample.

Distance, μm	0	5	10	15	20	25	30
*C*, GPa	99.2	91.8	98	161	154.1	142.7	130.8
$E_{r}$, GPa	211.36	198.6	205.8	231.8	224.36	218.38	206.33
*E*, GPa	235.0	217.9	227.5	263.4	252.9	244.6	228.2
$\sigma_{0.033}$, MPa	1268.71	1169.02	1259.85	2443.72	2326.34	2098.89	1894.36
*n*	0.37	0.49	0.47	0.36	0.33	0.41	0.35
$\sigma_{y}$, MPa	421.68	244.67	246.23	1132.3	1157.44	782.04	852.4
$\varepsilon_{y}$, %	0.18	0.11	0.11	0.43	0.46	0.32	0.37
*R_p0.2_*	593.26	439.27	455.91	1361.97	1382.72	1028.84	1037.52

**Table 3:** HAGB density, LAGB density, Σ3 TB density, and GND density statistics of the localized interfacial region.

	HAGB, ×10^12^/m^2^	LAGB, ×10^12^/m^2^	Σ3, ×10^12^/m^2^	Avg. GND, ×10^14^/m^2^
Weathering steel	0.4117	0.124	0.02395	4.12
Interface	1.15952	0.783	0.42	12.29
Stainless steel	0.1869	0.28372	0.0177	7.28

**Table 4:** Diffusion extent in different samples.

Element	Cr		Ni		Mo	
	Distance, μm	*k*	Distance, μm	*k*	Distance, μm	*k*
R	9	2.38	1.4	4.35	2.9	0.68
FC	11.5	1.84	5.3	1.73	4.5	0.30
AC	7	2.81	4.6	2.18	4.6	0.40
WC	7.3	2.75	3.2	3.17	2.6	0.63

## Data Availability

The data that support the findings of this study are available from the corresponding authors upon reasonable request.

## References

[1] Ban H., Mei Y., Shi Y.J. (2021). Research advances of stainless-clad bimetallic steel structures. J. Eng. Mech..

[2] Wang S., Chen B., Chen C.X., Feng J.H., Yin F.X. (2018). Microstructure, mechanical properties and interface bonding mechanism of hot-rolled stainless steel clad plates at different rolling reduction ratios. J. Alloys Compd..

[3] Yan Z., Sun C., Liu S., Chang X., Tong W. (2025). Vacuum diffusion bonding strengthening mechanical properties of 304 stainless steel/low carbon steel composites by in-situ eutectic reaction. Vacuum.

[4] Liu B., Wang S., Fang W., Ma J., Yin F., He J., Feng J., Chen C. (2018). Microstructure and mechanical properties of hot rolled stainless steel clad plate by heat treatment. Mater. Chem. Phys..

[5] Ding Y., Cao R., Yan Y. (2020). Effects of heat treatment on fracture mechanism of martensite/austenite MLS composite plates by hot roll bonding. Mater. Sci. Eng. A.

[6] Ji Q., Li Y., Ye P., Fu W., Cao G., Han Q., Li X., Wu H., Fan G. (2025). The effect of the interface structure on the interfacial bonding strength of Ti/Al clad plates. Prog. Nat. Sci. Mater. Int..

[7] Li H., Zhang L., Zhang B., Zhang Q. (2019). Microstructure characterization and mechanical properties of stainless steel clad plate. Materials.

[8] Wang K., Yu H., Tian Y., Zhu Z., Gao J., Li Q. (2022). Tailoring of interface microstructure and bonding property in 1Cr17/8Cr13MoV/1Cr17 stainless steel clad plate with Ni interlayer. Mater. Sci. Eng. A.

[9] Liu B., Wang S., Fang W., Yin F., Chen C. (2019). Meso and microscale clad interface characteristics of hot-rolled stainless steel clad plate. Mater. Charact..

[10] Jin J.-C., Cho S., Kim K., Sim H., Park B.G., Lee Y.-K. (2023). Microstructures and intergranular corrosion resistances of hot-rolled austenitic stainless steel clad plates. J. Mater. Res. Technol..

[11] Zhang Q., Li S., Li R., Zhang B. (2019). Multiscale comparison study of void closure law and mechanism in the bimetal roll-bonding process. Metals.

[12] Zak S., Trost C.O.W., Kreiml P., Cordill M.J. (2022). Accurate measurement of thin film mechanical properties using nanoindentation. J. Mater. Res..

[13] Nayebi B., Parvin N., Asl M.S., Motallebzadeh A., Shokouhimehr M. (2021). Nanostructural and nanoindentation characterization of ZrB2 ceramics toughened with in-situ synthesized ZrC. Int. J. Refract. Met. Hard Mater..

[14] Wu Y., Li Y., Luo S., Lu M., Zhou N., Wang D., Zhang G. (2020). Multiscale elastic anisotropy of a shale characterized by cross-scale big data nanoindentation. Int. J. Rock Mech. Min. Sci..

[15] Dao M., Chollacoop N., Van Vliet K.J., Venkatesh T.A., Suresh S. (2001). Computational modeling of the forward and reverse problems in instrumented sharp indentation. Acta Mater..

[16] Cao Y.P., Lu J. (2004). A new method to extract the plastic properties of metal materials from an instrumented spherical indentation loading curve. Acta Mater..

[17] Field J.S., Swain M.V. (1993). A simple predictive model for spherical indentation. J. Mater. Res..

[18] Herbert E.G., Pharr G.M., Oliver W.C., Lucas B.N., Hay J. (2001). On the measurement of stress–strain curves by spherical indentation. Thin Solid Film..

[19] Oliver W.C., Pharr G.M. (1992). An improved technique for determining hardness and elastic modulus using load and displacement sensing indentation experiments. J. Mater. Res..

[20] Kalidindi S.R., Pathak S. (2008). Determination of the effective zero-point and the extraction of spherical nanoindentation stress–strain curves. Acta Mater..

[21] Wang Y., Gao B., Wei W., Cao Y., Li Z. (2024). Effects of weathering bridge steel and cooling rate on the interfacial microstructure and mechanical properties of stainless steel clad plates. Ind. Constr..

[22] Park S.J., Heogh W., Yang J., Kang S., Jeong W., Lee H., Jang T.-S., Jung H.-D., Jahazi M., Han S.C. (2024). Meta-structure of amorphous-inspired 65.1Co28.2Cr5.3Mo lattices augmented by artificial intelligence. Adv. Compos. Hybrid Mater..

[23] (2021). Metallic Materials—Tensile Testing—Part 1: Method of Test at Room Temperature.

[24] Bodhankar P.M., Gurada C., Shinde S., Muthurajan H., Kumar V. (2015). Nanoindentation based fatigue analysis of semiconductor bridge (SCB) for mechanical reliability. J. Mater. Sci. Surf. Eng..

[25] Zhang Z.-N., Li Y.-L., Wu W.-P. (2022). Effects of loading rate and peak load on nanoindentation creep behavior of DD407Ni-base single crystal superalloy. Trans. Nonferrous Met. Soc. China.

[26] Long X., Li Y., Shen Z., Su Y., Gu T., Siow K.S. (2024). Review of uniqueness challenge in inverse analysis of nanoindentation. J. Manuf. Process.

[27] Zheng G., Wang L., Zhang Y., Sun D., Dong Q. (2023). Rolling-induced enhancement of strength in Mg-3Al-3Nd-0.5Mn alloy. Mater. Lett..

[28] Liao Y., Song Y., Shu N., Niu Y., Zhang H., Sun B., Wang Y., Li C., Gu J. (2025). Enhanced strength-ductility synergy in ferrous medium-entropy alloys via single-step hot rolling. Mater. Sci. Eng. A.

[29] Li Y., Liang Z., Huang M. (2022). Strengthening contributions of dislocations and twins in warm-rolled TWIP steels. Int. J. Plast..

[30] Lin Z., Liu B., Yu W., Zhang B., Ji P., Feng J., Yin F. (2022). The evolution behavior and constitution characteristics of interfacial oxides in the hot-rolled stainless steel clad plate. Corros. Sci..

[31] Wang X., Embury J.D., Poole W.J., Esmaeili S., Lloyd D.J. (2003). Precipitation strengthening of the aluminum alloy AA6111. Met. Mater. Trans. A.

[32] Wang Z., Zhang W., Li M., Wu Z., Liang J., Zhang L. (2025). Comparison of high-strength low-alloy steels fabricated by wire arc additive manufacturing and conventional casting: Effect of quenching and tempering on microstructural evolution and mechanical properties. J. Alloys Compd..

[33] Vaughan M., Gerstein G., Harris R., Gibbons S., Barber R., Maier H., Karaman I. (2025). Effects of severe ausforming on hierarchical microstructural development and mechanical performance in a martensitic high-strength steel. Mater. Sci. Eng. A.

[34] Li Y., Chen X., An Y., Xie J., Zhang X., Cao W. (2024). Excellent combination of strength and ductility in austenitic lightweight steel achieved by warm rolling process. Mater. Sci. Eng. A.

[35] An Y., Chen X., Ren P., Cao W. (2022). Ultrastrong and ductile austenitic lightweight steel via ultra-fine grains and heterogeneous B2 precipitates. Mater. Sci. Eng. A.

[36] Meng Y., Gu X.-F., Zhang W.-Z. (2010). A study of a new type of deviation from the Kurdjumov–Sachs orientation relationship in face-centered-cubic/body-centered-cubic transformation system. Acta Mater..

[37] Tomida T., Wakita M., Yasuyama M., Sugaya S., Tomota Y., Vogel S. (2013). Memory effects of transformation textures in steel and its prediction by the double Kurdjumov–Sachs relation. Acta Mater..

[38] Wang Y., Gao B., Hu K. (2025). Effect of vacuum degree on interfacial microstructure and bonding property of 316L/Q420qENH clad plate. Iron Steel.

